# *SULT1A1* genetic polymorphisms and the association between smoking and oral cancer in a case-control study in Brazil

**DOI:** 10.3389/fonc.2012.00183

**Published:** 2012-12-18

**Authors:** Sabrina S. Santos, Rosalina J. Koifman, Rafaela M. Ferreira, Lilian F. Diniz, Paul Brennan, Paolo Boffetta, Sergio Koifman

**Affiliations:** ^1^National School of Public Health/FIOCRUZRio de Janeiro, Brazil; ^2^International Agency of Research on Cancer (IARC/WHO)Lyon, France; ^3^Institute for Translational Epidemiology and Tisch Cancer Institute, Mount Sinai School of MedicineNew York, NY, USA

**Keywords:** SULT 1A1 gene, polymorphisms, oral cancer, smoking, alcohol

## Abstract

**Introduction:** Oral cancer is a public health problem worldwide, being tobacco and alcohol consumption their main risk factors. Sulfotransferase (SULT) 1A1 (encoded by *SULT1A1*) is involved in procarcinogens metabolism, such as polycyclic aromatic hydrocarbons (PAHs) present in tobacco smoke. **Objective:** The aim of this study was to explore the magnitude of association between *SULT1A1* gene Arg^213^His polymorphism and oral cancer, and to explore the interaction between such polymorphism and smoking. **Methods:** A hospital-based case-control study was carried out in Rio de Janeiro, Brazil, during 1999–2002. Epidemiological data and biological samples were obtained from 202 oral cancer patients and 196 sex and age-frequency matched controls without cancer antecedents. **Results:** No association was observed between Arg^213^His *SULT1A1* polymorphism and oral cancer risk in overall analysis (OR = 1.06, 95% CI = 0.71–1.57). The magnitude of association between cigarette smoking and oral cancer was higher in individuals with a SULT1A1^*^1 isoform (wild type, genotype Arg/Arg) (OR = 10.19, 95% CI = 3.90–26.61) than in those with at least one SULT1A1^*^2 allele (genotypes Arg/His + His/His) (OR = 4.50, 95% CI =2.09–9.69). **Conclusion:** Our results suggest that Arg^213^His *SULT1A1* polymorphism may modulate the association between smoking and oral cancer. However, this association needs to be replicated in other studies: due to modest number of cases and controls, the role of chance in the observed association cannot be ruled out.

## Introduction

Oral cancer is a major public health problem worldwide, resulting in 263,020 new cases (3.8 cases/100,000) and 127,654 deaths (1.9/100,000) per year, with the highest incidence and mortality rates occurring in developing countries (Ferlay et al., 2010). In Brazil, according to 2012 estimates, oral cavity cancer will result in 9990 new cases in males (10 cases per 100,000 men) and 4180 in females (4/100,000 women). However, a marked regional variation is expected, with incidence rates ranging from 3/100,000 men (2/100,000 women) in the North region to 15/100,000 men (6/100,000 women) in the Southeast (INCA, 2011).

Tobacco and alcohol consumption are described as the major environmental risk factors to oral cancer (Anantharaman et al., 2011; Lubin et al., 2011). Other environmental factors are diet, pollution (Johnson et al., 2011), oral hygiene (Guha et al., 2007), and certain virus strains, such as human papillomavirus (HPV) (Lambert et al., 2011). Regarding genetic risk factors, some authors have described an association between xenobiotic metabolizing enzymes polymorphisms and the development of oral cancer (Jefferies et al., 1999; Tripathy and Roy, 2006). However, the basis of this genetic susceptibility is not well understood (Ram et al., 2011).

The sulfotransferases (SULTs) are phase II detoxification enzymes involved in the biotransformation of a wide variety of xenobiotics and endogenous steroid (Nowell and Falany, 2006). The SULT1A1 isoform is involved in toxic substances inactivation, but it can also bioactive pro-carcinogens such as heterocyclic amines (HCAs) and polycyclic aromatic hydrocarbons (PAHs) (Bardakci et al., 2008; Koike et al., 2008), both present in tobacco smoke (International Agency for Research on Cancer: Tobacco smoke, 2004). *SULT1A1* is a polymorphic gene, and its Arg^213^His most studied polymorphism results from G to A nucleotide replacement at exon 7, generating SULT1A1^*^2 isoform (Nagar et al., 2006). The protein derived from SULT1A1^*^2 (^213^His variant allele), comparatively to that produced by the wild type (SULT1A1^*^1), has a twice lower catalytic activity and more reduced thermal stability (Koike et al., 2008).

SULT1A1^*^2 isoform is associated with increased risks of lung (Pachouri et al., 2006; Liao et al., 2012), stomach cancer (Liang et al., 2004; Boccia et al., 2005), urothelial cancer (Roupret et al., 2007), and breast cancer (Lee et al., 2012). However, others studies reported that SULT1A1^*^2 isoform could confer not statistically significant reduced risks to bladder cancer (Hung et al., 2004) and colorectal cancer (Nowell et al., 2002), as well as a statistically significant lung cancer risk reduction in heterozygous individuals (Ihsan et al., 2011).

With regard to oral cancer, SULT1A1^*^2 isoform has only been studied in Taiwan. No association between Arg^213^His *SULT1A1* polymorphisms and oral cancer was observed in that study, but oral cancer risk in betel quid chewers and smokers seemed to be lower in individuals with a SULT1A1^*^2 isoform compared to those with the wild type genotype (Chung et al., 2009).

The aim of this study was to investigate the association between Arg^213^His *SULT1A1* gene polymorphism and oral cancer, and to explore any interaction between this polymorphism and smoking with regard to oral cancer risk.

## Methods

This investigation is part of a multicentric hospital-based case-control study carried out in Brazil, Argentina, and Cuba aiming to explore the association between several environmental and genetic risk factors with oral, larynx, and esophageal cancers (Guha et al., 2007). The city of Rio de Janeiro was one of the places wherein the study was performed during 1999–2002, and as previously described (Marques et al., 2006), cases were 202 patients between 15 and 79-year-old with an histopathological confirmed diagnosis of oral cavity squamous cell carcinoma without previous treatment. Cases were diagnosed in Rio de Janeiro at the public and free care coverage Brazilian National Cancer Institute (INCA). Controls (196 patients) were gender and age-frequency matched to cases, being enrolled among hospitalized patients with no-neoplastic diseases (alcohol- or tobacco-related illnesses excluded) in two public general hospitals, the Institute of Trauma—Orthopaedics (INTO) and the Souza Aguiar Municipality General Hospital, both offering free and universal care in the same city. Hospitalization causes distribution among controls were: injury, poisoning, and certain other consequences of external causes 28.5%; digestive system diseases, 19.4%; genitourinary system diseases, 17.4%; musculoskeletal system and connective tissue diseases, 12.8%; respiratory system diseases, 8.7%; infectious and parasitic diseases, 6.1%; skin and subcutaneous tissue diseases, 2.6%; and 0.5%, others.

Therefore, control group included a variety of ill individuals enrolled in general hospitals, all of them presenting illnesses unrelated to smoking and alcohol intake in their natural history (i.e., emphysema, alcoholic cirrhosis, and others).

All participants were residents in the Metropolitan Region of Rio de Janeiro. Trained interviewers conducted in-person interviews to elicit information on demographic background, tobacco, and alcohol consumption and other lifestyle habits. Participating rate was 95% for cases and 86% for controls.

Peripheral blood samples collected in EDTA Vacutainer tubes were used for genomic DNA extraction following a standard protocol (Lahiri and Nurnberger, 1991). All proceedings were approved by the Ethics Research Committees of all involved institutions.

Genetic polymorphisms were assessed by previously described PCR-RFLP protocols (Coughtrie et al., 1999), with minor modifications. In brief, the amplification of target DNA was achieved by PCR optimized conditions as follows: a final reaction volume of 25 μL was composed of 100–200 ng of DNA, 0.2 mM of each dTNP, 4 mM of MgCl_2_, 0.75 U of Platinum Taq DNA polymerase (Invitrogen), 1× PCR buffer (Invitrogen), and 10 pmol of each primer (forward 5′gttggctctgcagggtttctagga3′ and reverse 5′cccaaacccccgtgctggccagcaccc3′). The reaction conditions used were: a pre-denaturation at 94°C for 5 min followed by 50 cycles with three steps each (94°C for 30 s, 68°C for 30 s, and 72°C for 45 s), and a cycle of 7 min at 72°C. Negative controls were included in every run, and the success of amplification was confirmed in agarose 1.5% gels, stained with Gel Red (Biotium), and visualized under ultraviolet (UV) light. Endonuclease digestions were performed as follows: a final reaction volume of 20 μL composed of 5 μL of PCR products, 5 U of *HaeII* enzyme (BioLabs), and 1× reactin buffer (BioLabs), using overnight 37°C incubation conditions. Determination of genotypes was performed in agarose 3% gels, visualized under UV light.

Goodness-of-fit of genotype distribution to Hardy–Weinberg equilibrium was ascertained for controls. Unconditional logistic regression models were used to calculate unadjusted and adjusted odds ratios (OR) and 95% confidence intervals (95% CI) for the association between *SULT1A1* polymorphism and oral cancer and to explore any interaction between this polymorphism and environmental risk factors. All statistical analyses were done using STATA 10.0 software.

## Results

The distribution of oral cancer cases and controls according to sex, age, skin color, smoking, alcohol consumption, and Arg^213^His *SULT1A1* genotype are presented at Table 1. About 82.7% of cases and 76.5% of controls were male. Mean age was 55.9 years (±9.9) among cases and 55.1 years (±11.7) among controls. Regarding skin color, whites accounted for 41.6% of cases and 53.6% of controls (*p* = 0.02). Smoking antecedents were more frequent among oral cancer cases than controls: among the former, 76.7% were smokers and 14.4% ex-smokers (respectively, 41.3 and 28.1% among controls, *p* < 0.01). Alcohol intake antecedents were reported by 67.3% of cases and 51.0% of controls (*p* < 0.01). Data analysis did not show an association between the presence of at least one SULT1A1^*^2 allele (genotypes Arg/His + His/His) and oral cancer (OR = 1.06, 95% CI 0.71–1.57). The OR adjustment for selected confounders (smoking, skin color, age, and sex) revealed similar results (OR = 1.07, 95% CI = 0.69–1.65).

**Table 1:** **Distribution of oral cancer cases and controls according to sex, age, skin color, smoking, alcohol intake, and Arg^213^His *SULT1A1* genotype, Rio de Janeiro, Brazil, 1999–2002**.

	**Variables**	**Controls *N* (%)**	**Cases *N* (%)**	**OR (95% Cl)**
Sex	Female	46 (23.47)	35 (17.33)	1.00
	Male	150 (76.53)	167 (82.67)	1.46 (0.89–2.39)
Age (year)	23–40	21 (10.71)	9 (4.46)	1.00
	41–50	50 (25.51)	54 (26.73)	2.52 (1.05–6.02)
	51–60	49 (25.00)	72 (35.64)	3.43 (1.45–8.11)
	61–79	76 (38.78)	67 (33.17)	2.06 (0.88–4.80)
Skin color	White	105 (53.57)	84 (41.58)	1.00
	Non-white	91 (46.43)	118 (58.42)	1.62 (1.09–2.41)
Smoking	Never smoker	60 (30.61)	18 (8.91)	1.00
	Current smoker	81 (41.33)	155 (76.73)	6.38 (3.53–11.52)
	Former smoker	55 (28.06)	29 (14.36)	1.76 (0.88–3.51)
Alcohol intake	No	53 (27.04)	13 (6.44)	1.00
	Current	100 (51.02)	136 (67.33)	5.54 (2.87–10.72)
	Former	43 (21.94)	53 (26.24)	5.02 (2.43–10.40)
*SULT1A1*	Arg/Arg	94 (47.96)	94 (46.53)	1.00
	Arg/His	82 (41.84)	89 (44.06)	1.08 (0.72–1.64)
	His/His	20 (10.20)	19 (9.41)	0.95 (0.48–1.89)
	Arg/His +			
	His/His	102 (52.04)	108 (53.47)	1.06 (0.71–1.57)^*^

* Adjusted for alcohol consumption, smoking, skin color, age, and sex.

The association between smoking and oral cancer according to Arg^213^His *SULT1A1* gene polymorphisms is presented at Table 2. Among subjects with an Arg/Arg genotype, an estimated 10-folds higher risk of developing oral cancer was observed among smokers (OR = 10.2, 95% CI = 3.90–26.61) comparatively to no-smokers. Among former smokers, the estimated risk was OR = 2.98 (95% CI = 1.01–8.78). Among individuals who had at least one SULT1A1^*^2 allele (genotypes Arg/His and His/His), the risk of oral cancer associated with smoking revealed an OR = 4.50 (95% CI = 2.09–9.69) for smokers, and OR = 1.17 (95% CI = 0.46–2.95) for former smokers. When adjusted by age, alcohol consumption, skin color, and sex, such heterogeneity between genotype groups became even higher (Table 2).

**Table 2:** **Association between smoking and oral cancer according to Arg^213^His *SULT1A1* polymorphism, oral cancer cases and controls, Rio de Janeiro, Brazil, 1999–2002**.

**Subject**	**Genotype OR (95% CI)**	
	**Arg/Arg**	**Arg/His + His/His**
Never smoker	1.00	1.00
Ever smoker	7.22 (2.84–18.33)	3.18 (1.52–6.65)
Current Smoker	10.19 (3.90–26.61)	4.50 (2.09–9.69)
Ex-smoker	2.98 (1.01–8.78)	1.17 (0.46–2.95)

OR_interaction_, 0.44; p, 0.176.

## Discussion

SULT1A1 is a phase II enzyme involved in the metabolism of a wide variety of xenobiotics and pro-carcinogens bioactivation, such as PAHs as benzopyrene, which are present in cigarette smoke (Bardakci et al., 2008).

Several epidemiological studies have consistently shown tobacco smoking as a major oral cancer risk factor (Garrote et al., 2001; Lubin et al., 2011). Moreover, a marked oral cancer risk reduction has been observed after quitting smoking, thus highlighting the role carcinogenicity of tobacco smoke to oral cavity (Castellsagué et al., 2004).

Genetic polymorphisms participating in cigarette compounds metabolism could affect individual cancer susceptibility by altering the enzymatic expression or function, which would result in increased or decreased carcinogens activation. The reactive intermediate metabolites formed during smoke compounds metabolism, if not eliminated, can eventually form covalent reactions (adducts) with DNA, RNA, or proteins. Therefore, extensive DNA damage may occur, increasing cancer risk (Miller et al., 2001).

Thus, although tobacco carcinogens can promote a variety of genetic alterations alone, there is evidence of the involvement of xenobiotics metabolism genes in the process of transformation of benign oral lesions into oral carcinomas (Lichtenstein et al., 2000).

Since SULT1A1 is involved in bioactivation of tobacco smoke pro-carcinogens and smoking is a major oral cancer risk factor, Arg^213^His *SULT1A1* polymorphism can hypothetically be associated with oral carcinogenesis in smokers. In our study, the magnitude of association between cigarette smoking and oral cancer was higher in individuals with a SULT1A1^*^1 isoform (wild type, genotype Arg/Arg) (OR = 10.19, 95% CI = 3.90–26.61) than in individuals with at least one SULT1A1^*^2 allele (genotypes Arg/His + His/His) (OR = 4.50 95% CI = 2.09–9.69), (Table 2, OR interaction *p* > 0.05). These results suggest that the low enzyme activity variant SULT1A1^*^2 (^213^His) (Koike et al., 2008) may lead to a decreased smoking procarcinogens (such as PAHs) bioactivation, thereby reducing the risk of smoking-related cancers. However, considering the small studied sample size, the occurrence of chance as an explanatory reason for this association cannot be ruled out. Additionally, it could also result from other unanalyzed *SULT1A1* polymorphisms, or other involved genes in cigarette smoke pro-carcinogens metabolism. In a small sample size case-control study, Chung et al. (2009) explored the association between *SUTT1A1* polymorphisms and oral squamous cell carcinoma (OSCC) susceptibility in male Taiwanese. They also reported that the presence of Arg^213^His *SULT1A1* polymorphisms was not associated with the risk of developing oral cancer (OR = 1.04, 95% CI = 0.19–5.12, when no other *SULT1A1* SNP was present). However, the risk of developing oral cancer in betel quid chewers and smokers seemed to be lower in SULT1A1^*^2 isoform patients comparatively to those with a wild type (OR = 0.58, 95% CI = 0.15–2.28).

The association between Arg^213^His *SULT1A1* polymorphism and other cancer types has been mixed (Wang et al., 2003). SULT1A1^*^2 (^213^His) allele has been associated with an increased risk of prostate cancer (OR = 1.60, 95% CI = 0.46–5.62) (Nowell et al., 2004), stomach cancer (OR = 3.32; 95% CI = 1.17–9.45) (Liang et al., 2004; Boccia et al., 2005), urothelial cancer (OR = 2.18, 95% CI = 1.28–3.69) (Roupret et al., 2007), and breast cancer (OR = 1.12, 95% CI = 1.02–1.24). On the other hand, a statistically non-significant reduced risks of bladder cancer (OR = 0.67, 95% CI = 0.45–1.03) (Hung et al., 2004) and colorectal cancer (OR = 0.6, 95% CI = 0.3–1.1) (Nowell et al., 2002) have been reported. With regard to lung cancer, Pachouri et al. (2006) reported an increased risk of lung cancer associated with His/His (SULT1A1^*^2 isoform) genotype (OR = 1.40, 95% CI = 0.48–4.06), which was higher among smokers (OR = 3.9, 95% CI = 1.99–7.81). In contrast, Ihsan et al. (2011) found an inverse association between *SULT1A1* Arg^213^His heterozygous genotype (Arg/His) and lung cancer (OR = 0.51, 95% CI = 0.33–0.78).

Results of this study suggest that Arg^213^His *SULT1A1* polymorphism may modulate susceptibility to oral cancer in smokers. However, as this study had a modest sample size, the role of chance cannot be excluded. Another limitation of this study was that we investigated a single polymorphism with limited data on smoking. Therefore, these results need to be replicated in further studies. Due to heterogeneous results on the role of Arg^213^His *SULT1A1* polymorphism in the activation of smoke procarcinogens and the consequent susceptibility to various cancers, GWAS exploring multiple genes involved in the metabolism of tobacco smoke compounds are needed to obtain more comprehensive evidence of possible interactions between these genes and smoking with regard to cancer risk.

### Conflict of interest statement

The authors declare that the research was conducted in the absence of any commercial or financial relationships that could be construed as a potential conflict of interest.
